# Characteristics and emergency mitigation of the 2018 Laochang landslide in Tianquan County, Sichuan Province, China

**DOI:** 10.1038/s41598-021-81337-x

**Published:** 2021-01-15

**Authors:** Zhuo Chen, Danqing Song, Lihu Dong

**Affiliations:** 1 Faculty of Geosciences and Environmental Engineering, Southwest Jiaotong University, Chengdu, 611756 China; 2 State Key Laboratory of Hydraulic and Mountain River Engineering, College of Water Resource and Hydropower, Sichuan University, Chengdu, 610065 China; 3 Department of Hydraulic Engineering, State Key Laboratory of Hydroscience and Engineering, Tsinghua University, Beijing, 100084 China; 4 School of Electrical Engineering, Shenyang University of Technology, Shenyang, 110870 China

**Keywords:** Natural hazards, Environmental sciences

## Abstract

This paper describes a recent landslide event, which occurred at Liucheng village in Tianquan County, Sichuan Province, China, on July 15, 2018. The Laochang landslide described in this research is an outstanding and valuable reference for understanding the characteristics of such kind of landslides that are geologically similar to the landslide. The deformation characteristics of the landslide are investigated based on field investigations, drilled boreholes, and exploratory trenches. The 225 residents of 64 households living on the flat platform were threatened by the landslide. Therefore, to guarantee the safety of human life and property becomes the primary emergency task. The anti-sliding piles were taken to stabilize the landslide and mitigate impacts caused by the landslide. Based on the analysis of the monitoring data, the effectiveness of anti-sliding piles is evaluated. The results indicate that the anti-sliding piles are effective in increasing the stability of the landslide, and this work can provide a reference for similar slope engineering projects.

## Introduction

Landslides represent a common geohazard in many parts of the world and became a major concern to national and local authorities^1, 2^. A large number of cases have been reported in many countries such as China, India, Vietnam, Japan^3–8^. Landslides in Southwest China are particularly widespread, threatening human activities and producing socio-economic losses^9–15^. Tianquan County is one of the landslide-prone areas in southwest Sichuan Province, China. The number of landslides has increased markedly since the 1970s, and these landslides have caused a lot of property damages each year. According to a 2018 survey, there were more than 220 landslides in Tianquan County, including more than 20 disastrous landslides.

**Figure 1:**
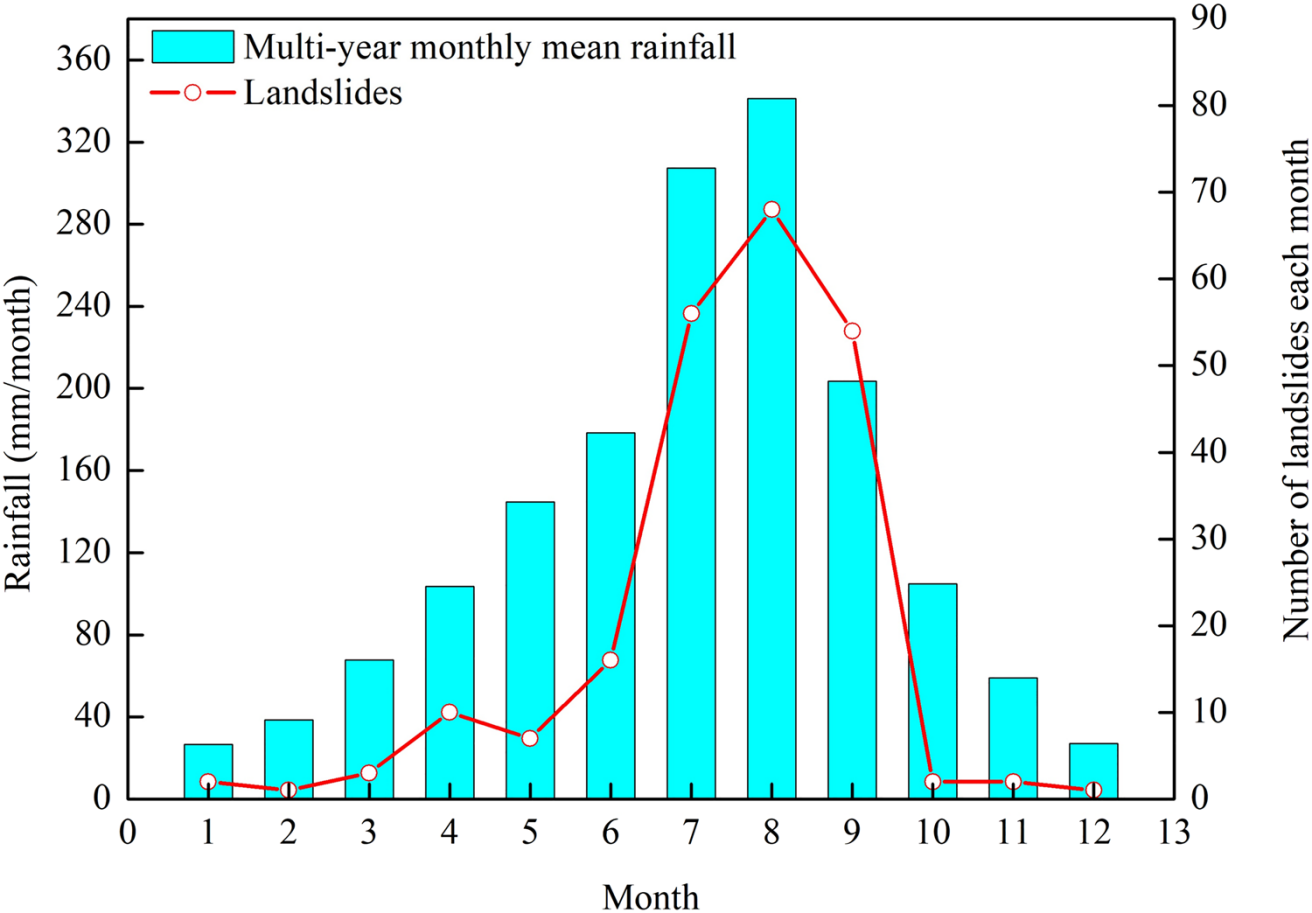
Graph showing the monthly distribution of the landslides and rainfall in the period 1971–2018 in Tianquan County.

**Figure 2:**
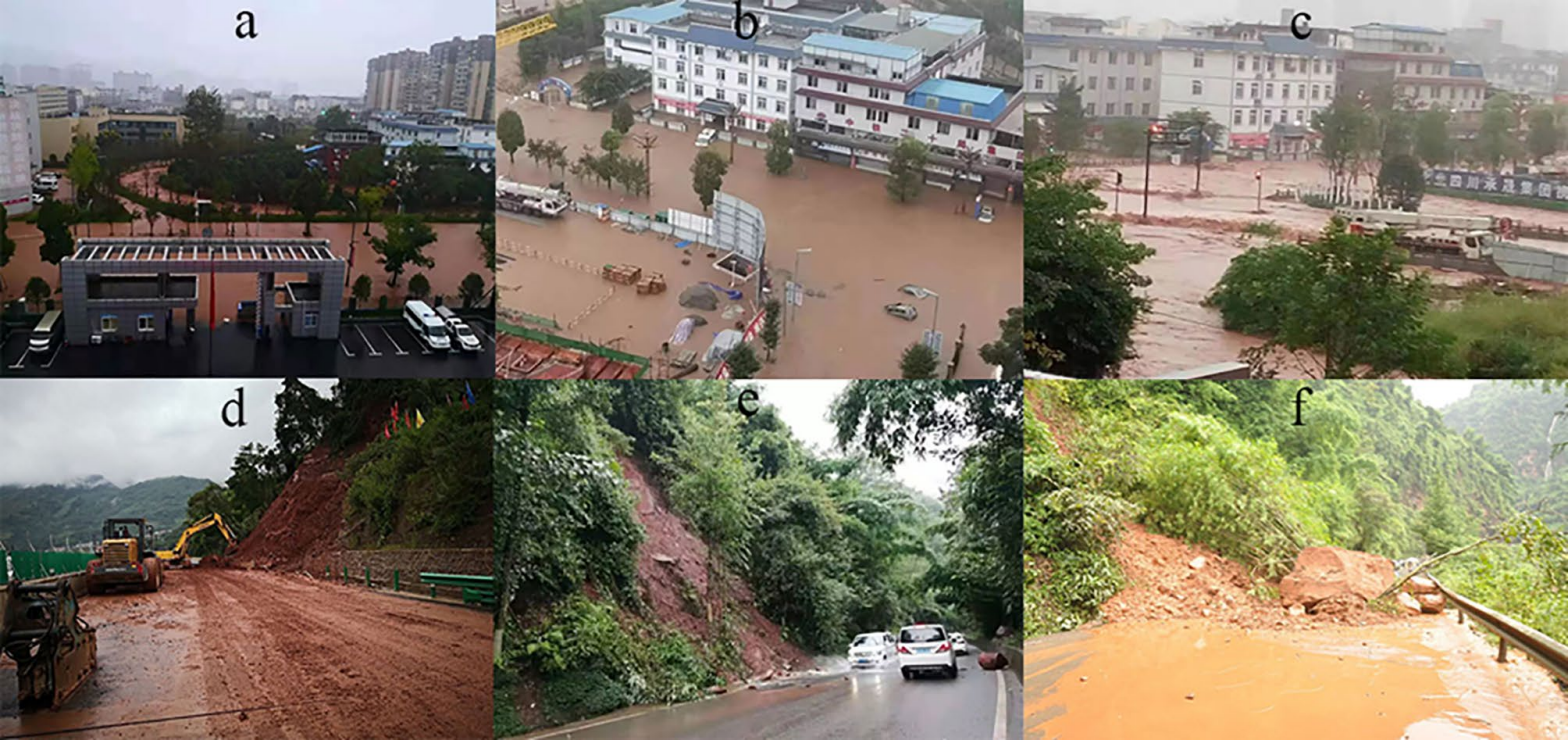
Photos of the rainfall-triggered flood and landslides in Tianquan County. (**a**–**c**) flood; (**d**–**f**) landslides.

The frequently occurred landslides in remote mountainous areas are receiving increasing attention from many researchers, because some of them may have devastating effects^16–23^. Landslides in these areas often cluster regionally with substantial negative consequences, posing important engineering problems. There are many factors such as intense or prolonged rainfall, earthquakes, and human activities (such as unplanned vertical cut, and irrigation) responsible for exacerbating hazardous situations in Tianquan County. As shown in Fig. 1, 87.39% of the recorded landslide events in the area occur between June and September, presenting a high correlation between the temporal distribution of the landslides and rainfall. During the summer seasons of 2010–2011 and 2013–2014, rainfall events affected most areas of the region, resulting in widespread landslides and floods (Fig. 2). Two major earthquakes occurred in the past 15 years: the 2008 Ms 8.0 Wenchuan earthquake (approximately 111.611 km away from the study area) and the 2013 Ms 7.0 Lushan earthquake (approximately 25.275 km away from the study area). Hovius et al. (2011) and Marc et al. (2015) found that for large earthquakes (6.6 ≤ Ms ≤ 7.6) direct effects on landslide rates can persist as long as 4–6 years^24, 25^. Therefore, the delayed effect of the two large earthquakes cannot be ignored. The repeated vibrations of earthquakes in this area increased the crack connectivity and weakened natural slopes, providing favorable conditions for slope failure.

In recent decades, urbanization has brought human settlements within reach of landslide hazards. Generally speaking, new urban areas are built in nearby areas at slightly higher elevations. However, land shortage for agriculture and construction has become a thorny problem in Southwest China, and thus some buildings have to be erected in the proximity of or within the landslide bodies. The building damage caused by landslides has increased because of the rapid development of urbanization on landslide-prone slopes in Tianquan County. In addition, many landslides also seem to be linked with the land conversion from natural forest to dry land agriculture in Tianquan County. Changes in land use impose an impact on the hydrological response of an area. Due to the high proportion of farmland, agricultural irrigation has become an important agent to trigger landslides. Therefore, investigation and mitigation of such landslide disasters have become a crucial aspect for guaranteeing the safety of human life and property.

A typical case study from the Laochang landslide located in the eastern sector of Tianquan County is presented. The Laochang landslide directly threatened the safety of 247 residents in 64 households, with potential economic losses exceeding 12 million CNY. This landslide is regionally outstanding for its dimensions and well-developed landslide morphology. Presently, slow continual deformation of the Laochang landslide can be easily identified as a sign that the landslide remains unstable. Effective engineering measures should be carried out to control the continuous displacement of the landslide. Authorized by the stakeholder, detailed field investigation, boreholes, and exploratory trenches were carried out to characterize the landslide features. Then the stabilization measure of the landslide is presented. The results of this study could provide useful guidance for the prevention and reduction of landslide hazard and risk in Tianquan County.

## Regional setting

**Figure 3:**
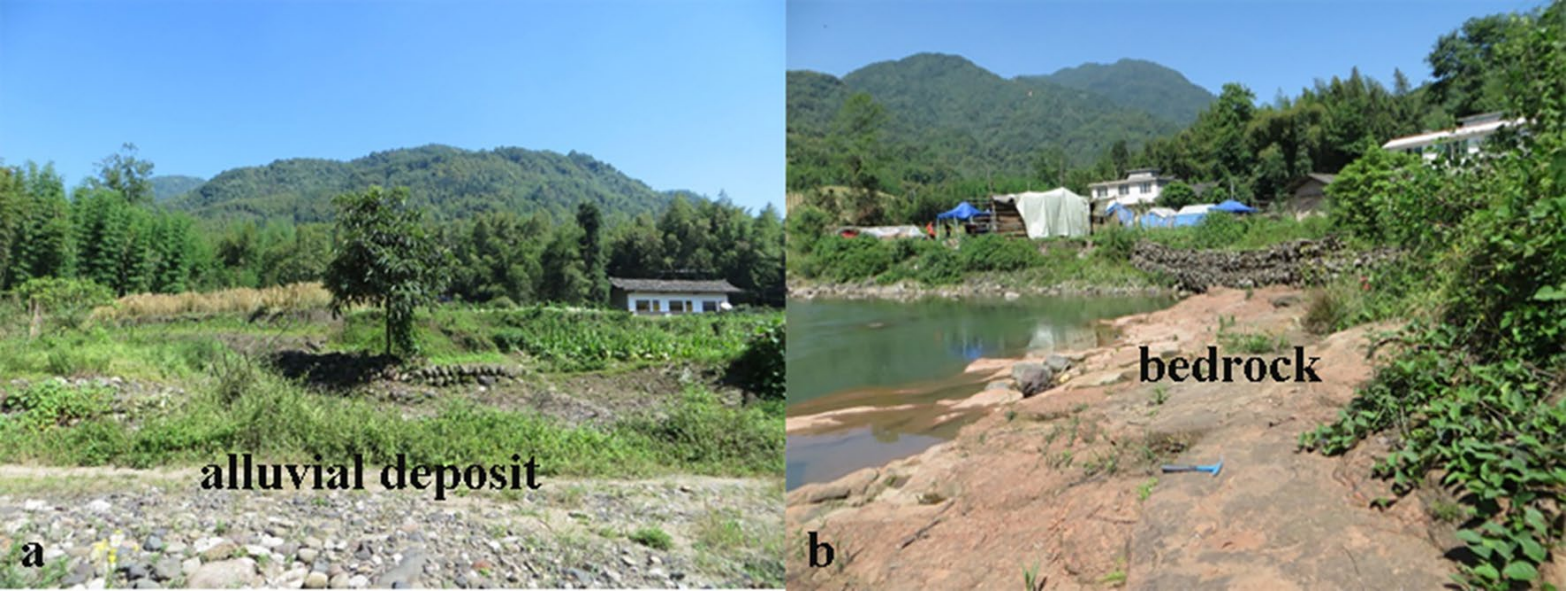
Bedrock and alluvial deposits of the study area.

The landslide is located at Liucheng village, Tianquan County, Sichuan Province, China, on the left bank of the Laochang River, a tributary of the Baoxing River. Liucheng village is situated in the eastern sector of Tianquan County, about 172 km far from Chengdu city, the capital of Sichuan Province. The study area is located in the east wing of Baoxing anticline. The nearest active fault to the Laochang landslide is the Shuangshi fault, about 6.6 km from the landslide boundary. The area is part of the transitional zone that stretches from the eastern margin of the Tibetan Plateau to the eastern Sichuan Basin. The terrain is characterized by low mountains, hills, and valleys with altitude ranging from 700 to 900 m above sea level. The exposed strata in this region include: (1) quaternary sediments, which consist of alluvial, colluvial, and residual deposits, as well as reworked landslide deposits; and (2) the Paleogene minshan group (E_*1-2*_* mn*), which consists of silty mudstone intercalated with thin siltstone (Fig. 3). According to the Ya’an Meteorological Bureau (http://sc.cma.gov.cn/ds/ya/) in Sichuan Province, the study area has a subtropical monsoon climate with an average annual temperature of 15.1 °C. The mean annual rainfall from 1971 to 2018 is 1,602.7 mm, and 64.30% of the annual average rainfall occurs between June and September.

## Characteristics of the Laochang landslide

**Figure 4:**
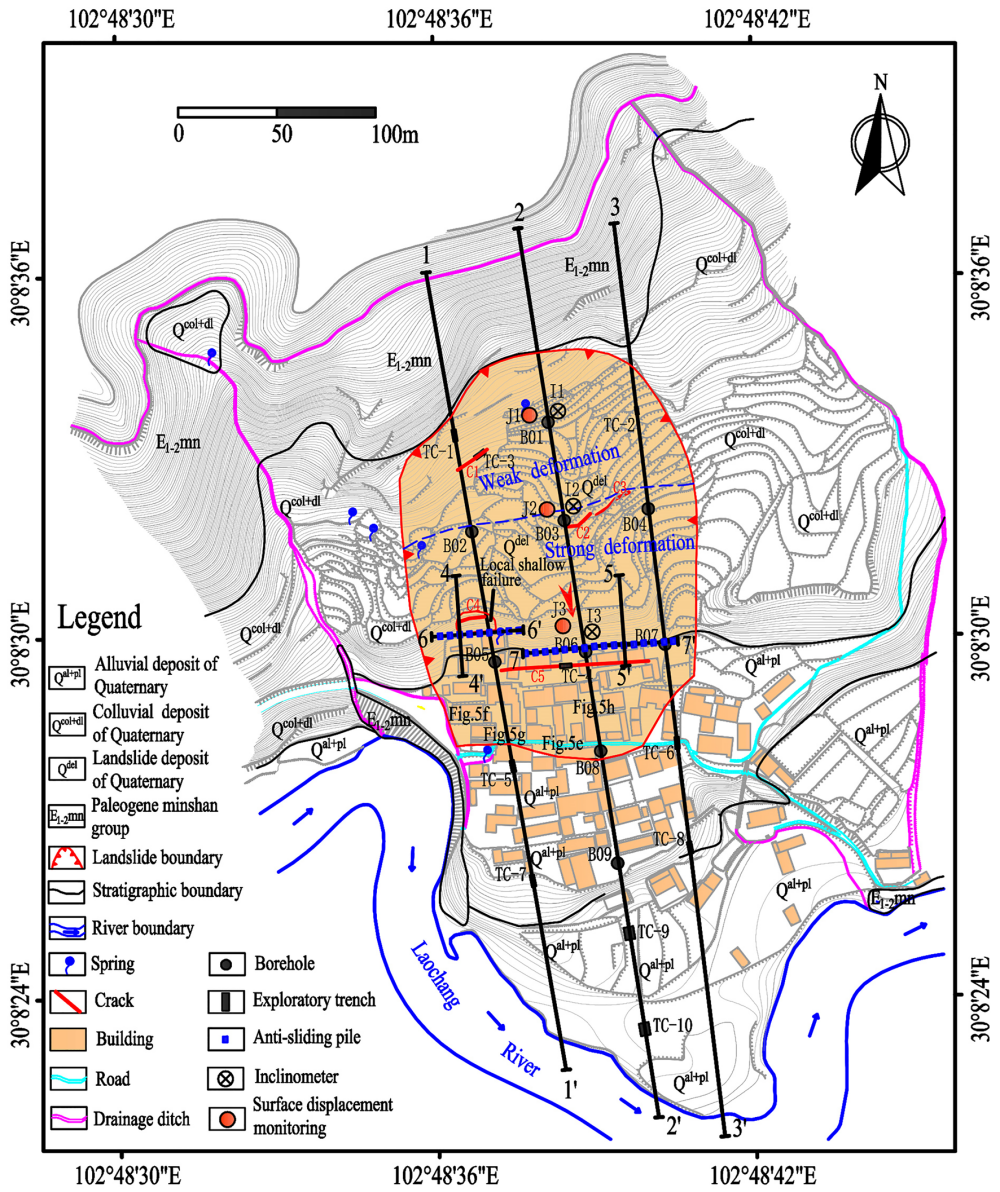
Topographic map of the landslide. The figure locations show the locations of Fig. 5.

The Laochang landslide (30° 8′ 31.76″ N, 102° 48′ 38.11″ E) has an irregular dustpan shape with a length of 210 m, a width of 150 m, and an average thickness of 10 m (Fig. 4). The area of the recent landslide is 3.15 × 10^4^ m^2^, the estimated volume is 3.15 × 10^5^ m^3^, the sliding direction is 171°, the mean slope gradient is 10°, the elevation is 753.5–791.5 m, and the relative elevation difference is 38 m. The lower slope is comparatively gentler than the upper part. The rural road traverses the anterior section of the landslide, and the Laochang River is situated in the leading edge of the landslide. Many houses stand around the landslide, at the foot of the slope. The drainage ditch is built in the proximity of the landslide.

**Figure 5:**
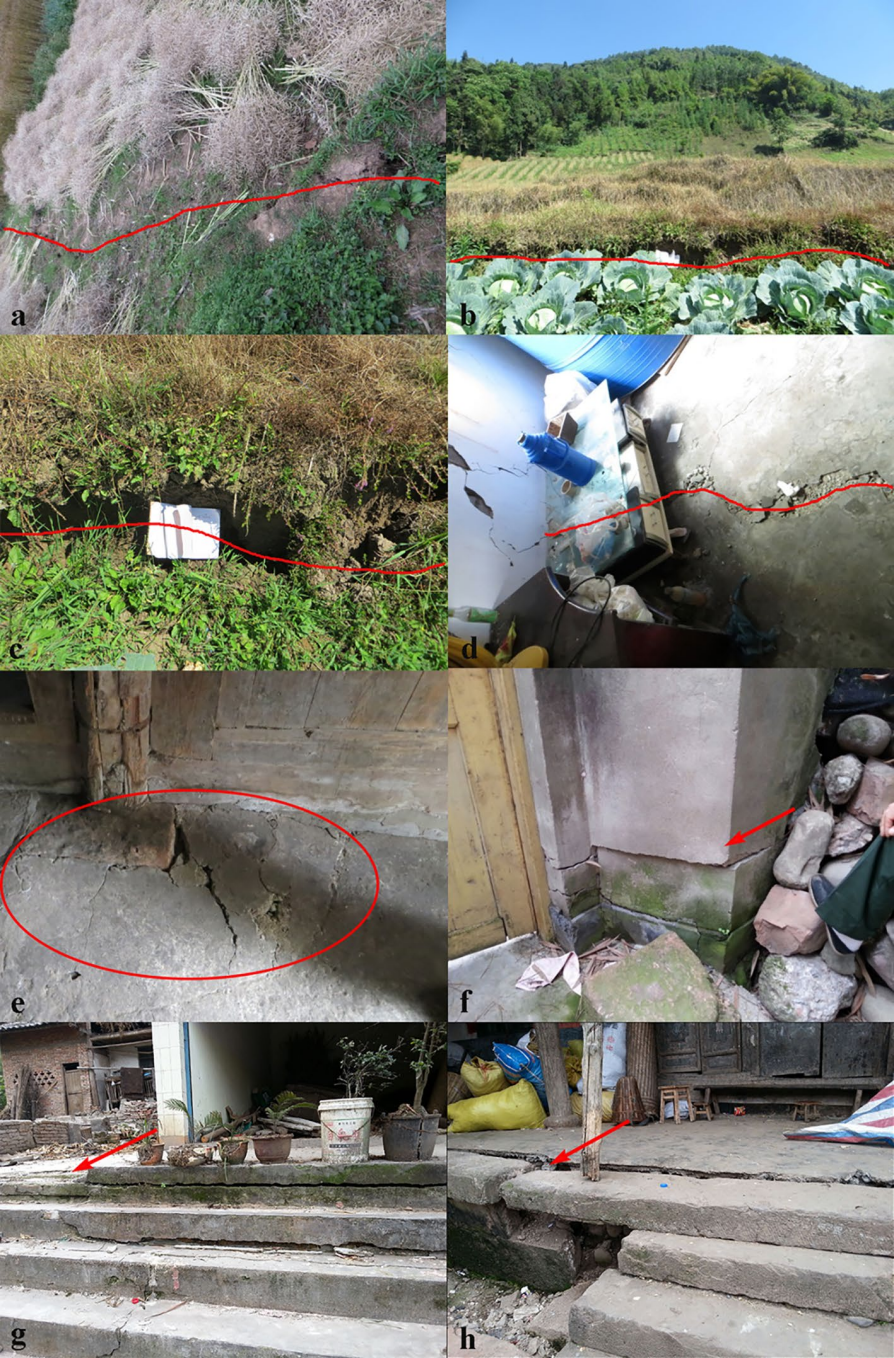
Macroscopic deformations have been observed within the landslides body. (**a**) Crack C1. (**b**) Crack C2. (**c**) Crack C3. (**d**) Crack C5. The locations of bulges and dislocations are shown in Fig. 4.

Macroscopic deformations, including cracks, minor scarps, bulges, and dislocations, have been observed within the landslide body (Fig. 5). Detailed site investigations revealed five cracks developed on the landslide surface. These cracks have widths of 7–30 cm, lengths of 15–63 m, vertical displacements of 12–50 cm, and observed depths of 30–80 cm, and they cause damage to several houses. The Wenchuan earthquake exerted a very intense impact in the landslide area, and four cracks (crack 1, crack 2, crack 3 and crack 5) developed on the landslide surface were caused by this earthquake (oral information from the local residents). According to the local residents, the Lushan earthquake enlarged these cracks. The extruding mass movement produced bulging cracks at the landslide toe (Fig. 5e). Severe structural damage was observed, resulting in the tilt of some houses (Fig. 5f–h).

**Figure 6:**
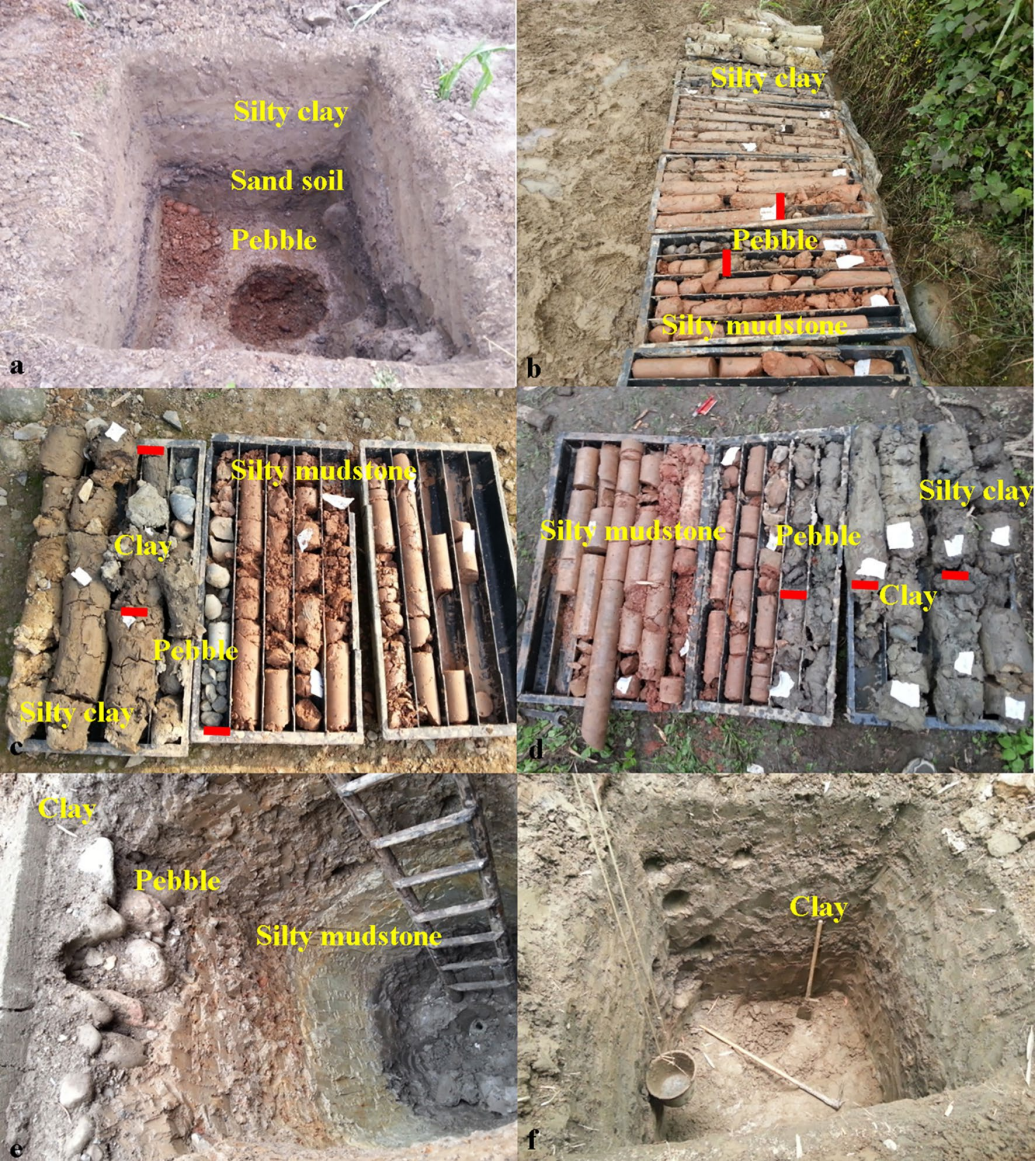
Lithological stratigraphic logs of boreholes and exploratory trenches. (**a**) TC-9. (**b**) B03. (**c**) B05. (**d**) B06. (**e**) TC-4. (**f**) TC-7.

**Figure 7:**
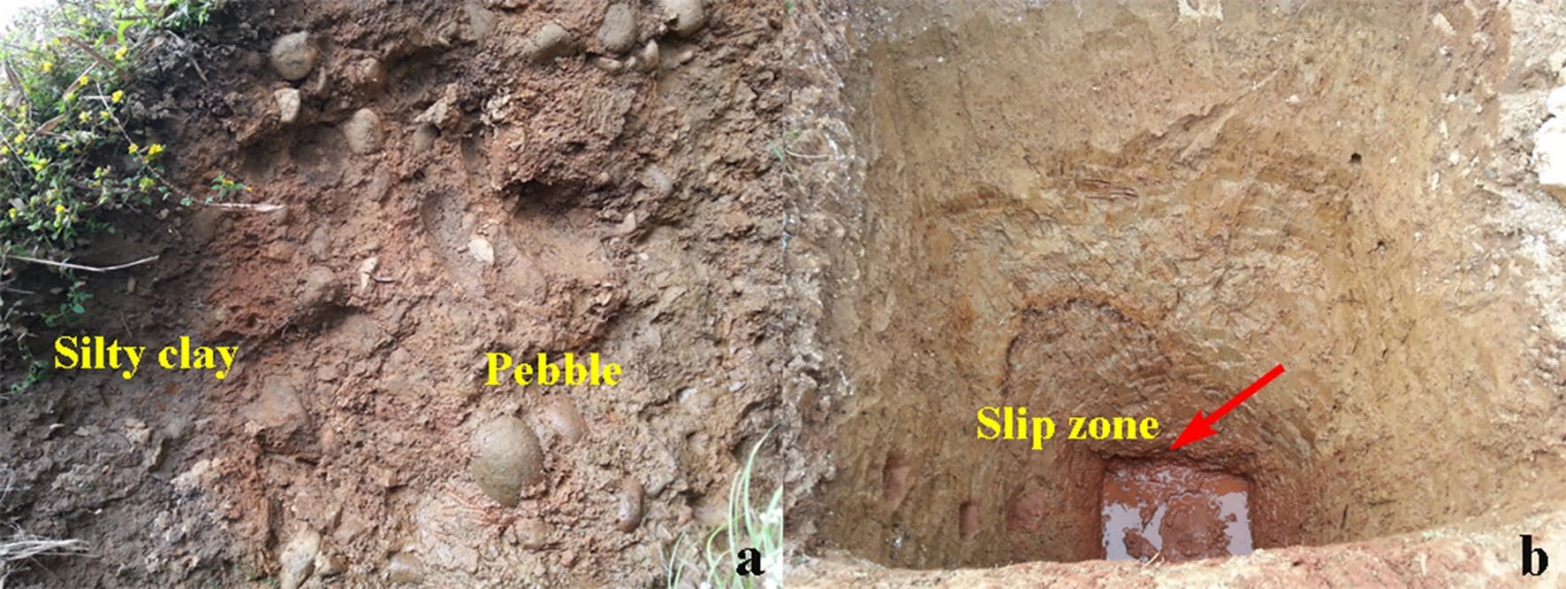
The structure of the sliding mass and the slip zone of the landslide exposed by exploratory trench.

**Figure 8:**
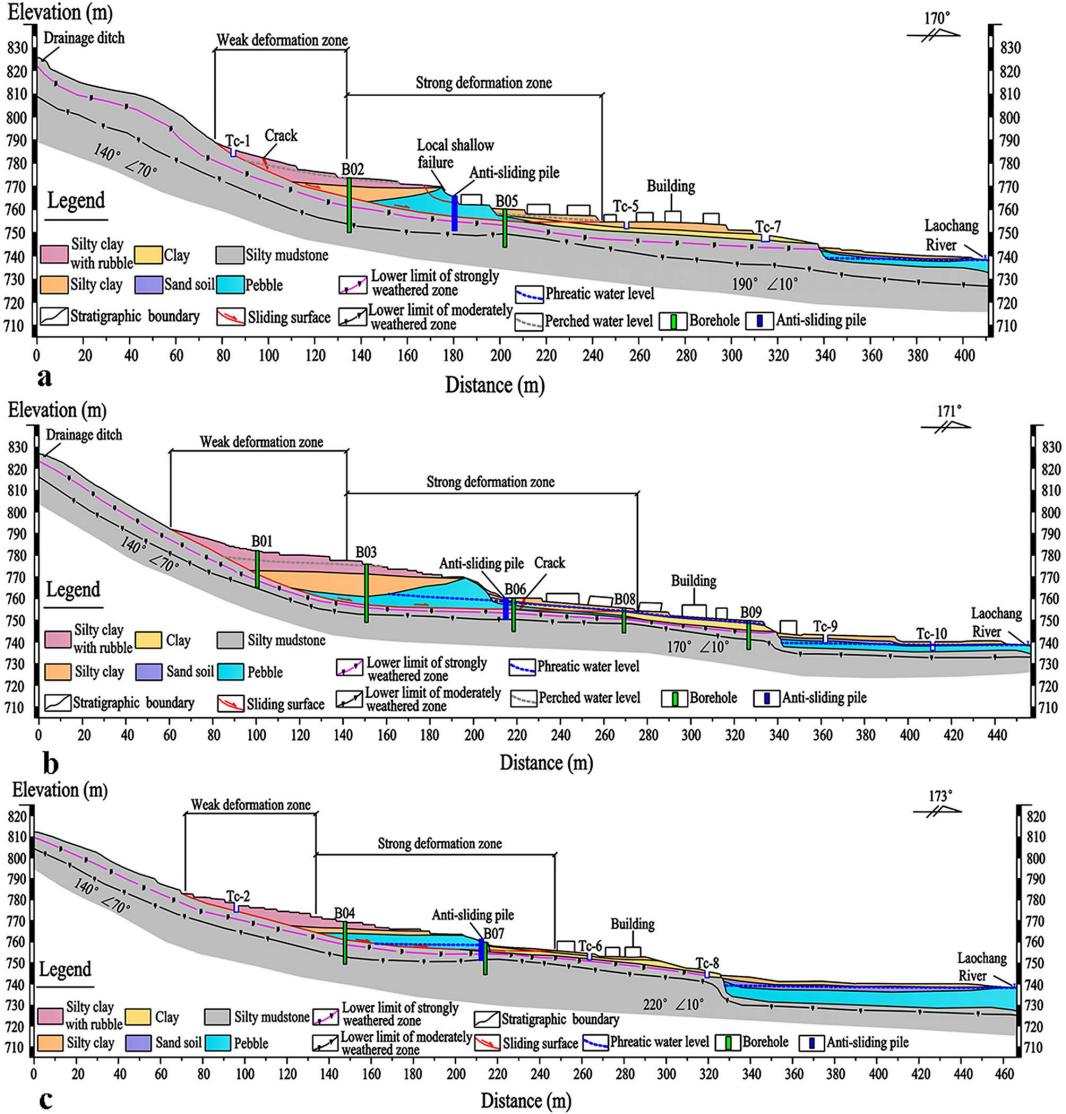
Longitudinal geological profiles: (**a**) 1–1′, (**b**) 2–2′, (**c**) 3–3′ (Fig. 4).

The geological structure of the landslide and the potential slip surface is obtained through boreholes and exploratory trenches, as shown in Figs. 6 and 7. Figure 4 shows the locations of the boreholes and the exploratory trenches. The landslide structure from bottom to top is pebble, silty clay, silty clay with rubble, respectively. According to the result of boreholes and the exploratory trenches, the location of the slip surface was confirmed (Figs. 7 and 8). The slip surface has a depth of approximately 3.8–15.8 m and is located at the interface between the superficial deposits and the underlying bedrock. The sliding zone is about 0.6–0.9 m thick, which is mainly composed of clay and small gravels.

According to field investigations and its deformation characteristics, the Laochang landslide can be roughly divided into two parts: the front strong deformation part and the rear weak deformation part (Figs. 4 and 8). Tensile cracks, surface bulges, and dislocations could be found in the front strong deformation part. The area and volume of the strong deformation part are approximately 2.03 × 10^4^ m^2^ and 2.31 × 10^5^ m^3^, respectively. The elevation of this zone ranges from 753.5 to 773.4 m, the average slope is 7°, and the average thickness is 11.4 m. The deformation of the landslide promotes the development of tension cracks and bulges. Several houses have suffered considerable damages, and the most severely damaged buildings are located at the landslide toe. Moreover, a possible area of local instability is observed near the middle part of the landslide (Figs. 4 and 8). Some small surface fissures were observed during the rainy season, but no obvious large deformation was apparent. The elevation of the weak deformation part is between 773.4 and 791.5 m, the average thickness is 8.6 m, the average slope is 13°, the area is approximately 1.12 × 10^4^ m^2^, and the estimated volume is 0.84 × 10^5^ m^3^.

Figure 8 shows the landslide structure and the groundwater level in the landslide body. The groundwater in the landslide area mainly includes two types: the perched water and the phreatic water. The perched water is mainly found in the silty clay with rubble, and the phreatic water is found primarily in the contact zone between the silty mudstone and the overlying pebble. Tensile cracks observed on the landslide surface provide a preferential flow path for the rainwater infiltration. Continuous rainfall led to increased groundwater and thus decreased the effective stress in the slope. In addition, a significant amount of irrigation water in spring and summer also increased the groundwater level, exerting a negative effect on slope stability. Some of the groundwater is discharged to the ground surface in the form of spring water, indicating the abundance of groundwater in the proximity of or within the landslide body. The high groundwater level reduces the resisting force and increases the sliding force, thereby facilitating the instability and deformation.

According to the local officials, the number of houses shows an increase from 7 houses in 1990 to 47 in 2018 in the landslide toe, indicating insufficient urban planning by the government. Slope cutting for houses, without slope support measures, caused disturbance to the slope and changed the stress distribution within the slope, and the resisting force of the anti-slip section decreased markedly. The modification of the landslide surface geometry via toe removal further reduced the slope stability. Many local residents claimed that they found a large and long crack in the middle part of the landslide in the 1990s. In 1991, a geological team also found that the width of this crack has developed to 0.5 m. The same witnesses stated that the landslide activity increased after the summer of 1998. The oral information of local residents and engineers indicates that the Laochang landslide has a long deformation history.

## Stability analysis of the Laochang landslide

In China, the limit equilibrium method^26^, preferred among many researchers, becomes the most commonly stability evaluation method of slopes due to its conceptual clarity and simple calculation process. It should be noticed, however, that this method is not appropriate when evaluating some special landslides, such as diffuse landslides plastic flow, lateral spreads, and the rockslide undergoing sudden brittle fracture^27^. The crucial prerequisites of this method involve three aspects: (1) the sliding plane has been determined; (2) the failure surface observes a Mohr–Coulomb failure criterion; and (3) the sliding body maintains coherent during sliding.

The selection of appropriate shear strength parameters is crucial for the accurate determination of slope stability. However, it is difficult to directly obtain the appropriate parameters from testing due to the heterogeneity of the rock and soil mass. The back-calculation approach provides a relatively easy way to do this, especially when slopes are under on-going failure^11, 28^. The mechanical parameters obtained from laboratory tests provided good references for later back-calculation. When the safety factor (*Fs*) is 1 (limit equilibrium state of slope stability) for back-calculation, the strength parameters of the slide zone soil can be calculated using the following equations:

1 $$C = \frac{{Fs\sum {W_{i} \sin \alpha_{i} - \tan \varphi \sum {W_{i} \cos \alpha_{i} } } }}{L}$$

2 $$\varphi = \arctan \left( {\frac{{Fs\sum {W_{i} \sin \alpha_{i} - CL} }}{{\sum {W_{i} \cos \alpha_{i} } }}} \right)$$

where *W*_*i*_ is the weight of the *i*th calculation block; *L* is the length of the slip surface; and *α*_*i*_ is the dip angle of the slip surface of the *i*th calculation block.

In practice, Chinese engineers have empirically confirmed sound relationships between the calculated safety factor and the deformation and failure features of the landslide (Table 1). The slip surface is presented as a polyline, and thus the transfer coefficient method is used to calculate the *Fs* of the slip surface. The *Fs* of the slip surface using the transfer coefficient method is calculated according to the following equations:

3 $$Fs = \frac{{\sum\limits_{i = 1}^{n - 1} {(((W_{i} ((1 - r_{U} )\cos \alpha_{i} - A\sin \alpha_{i} ) - R_{{D_{i} }} )\tan \varphi_{i} + C_{i} L_{i} )\prod\limits_{j = i}^{n - 1} {\psi_{j} ) + R_{n} } } }}{{\sum\limits_{i = 1}^{n - 1} {[(W_{i} (\sin \alpha_{i} + A\cos \alpha_{i} ) + T_{{D_{i} }} )\prod\limits_{j = i}^{n - 1} {\psi_{j} ] + T_{n} } } }}$$

4 $$T_{{D_{i} }} = \gamma_{W} h_{{i_{W} }} L_{i} \cos \alpha_{i} \sin \beta_{i} \cos (\alpha_{i} - \beta_{i} )$$

5 $$R_{{D_{i} }} = \gamma_{W} h_{{i_{W} }} L_{i} \cos \alpha_{i} \sin \beta_{i} \sin (\alpha_{i} - \beta_{i} )$$

6 $$R_{n} = (W_{n} ((1 - r_{U} )\cos \alpha_{n} - A\sin \alpha_{n} ) - R_{{D_{n} }} )\tan \varphi_{n} + C_{n} L_{n}$$

7 $$T_{n} = (W_{n} (\sin \alpha_{n} + A\cos \alpha_{n} ) + T_{{D_{n} }}$$

8 $$\prod\limits_{j = i}^{n - 1} {\psi_{j} = \psi_{i} \psi_{i + 1} \psi_{i + 2} \ldots \psi_{n - 1} }$$

9 $$\psi_{j} = \cos (\alpha_{i} - \alpha_{i + 1} ) - \sin (\alpha_{i} - \alpha_{i + 1} )\tan \varphi_{i + 1}$$

where *C*_*i*_ and *φ*_*i*_ are the cohesion and friction angle of the slip surface of the *i*th calculation block, respectively; *T*_*Di*_ and *R*_*Di*_ are the forces generated by the seepage pressure; *A* is the earthquake acceleration; *L*_*i*_ is the *i*th calculation block slip surface length; *β*_*i*_ is the groundwater flow direction of *i*th calculation block; *r*_*U*_ is the pore pressure ratio; *γ*_*W*_ is the unit weight of water; *h*_*iW*_ is the groundwater level of the *i*th calculation block; *ψ*_*j*_ is the transfer coefficient of the *i*th calculation block to the (*i* + 1)th block; *i* = 1, 2, …, *n*-1; and *n* is the total number of the soil blocks.

**Table 1:** Relationship between safety factor and developed stage^28^.

Stage	Surface deformation	Maximal displacement of sliding plane	Status of sliding plane	Safety factor
Embryonic	Invisible	Millimeter	–	≥ 1.1
Creep	Deformation evidence at scarp and toe	Centimeter	At limit equilibrium state	1.0–1.1
Sliding	Main scarp subsidence and toe bulge	Decimeter–meters	Complete failure	0.95–1.0
Emplacement	Sedimentation, partial collapse	–	–	≥ 1.0

Three cross-sections, including 1–1′, 2–2′, and 3–3′, are used in the stability analysis of the Laochang landslide. The average water table heights of the three sections are 2.54 m, 5.07 m, and 2.67 m, respectively. The peak ground acceleration (PGA) of 0.15 g is used for pseudo-static analysis. The physical and mechanical parameters obtained from laboratory tests are listed in Table 2. The safety factors of the Laochang landslide were calculated under three scenarios (Table 3), indicating that the landslide without an anti-sliding pile would become unstable under both strong rainfall and earthquake conditions.

**Table 2:** Physical and mechanical parameters of landslide mass and sliding zone soil.

Parameters	Landslide mass	Sliding zone
Natural unit gravity (kN/m^3^)	19.6	18.7
Saturated unit gravity (kN/m^3^)	20.5	19.2
Cohesion in natural state (kPa)	10.3	7.6
Cohesion in saturated state (kPa)	9.6	7.1
Friction angle in natural state (°)	29	7.7
Friction angle in saturated state (°)	19.3	7.4

**Table 3:** Safety factors of the Laochang landslide under different scenarios.

Typical sections	Anti-sliding pile	Scenarios	Safety factor	Stable state
1–1′	No	Normal	1.03	Basic stable
		Rainstorm	0.86	Unstable
		Earthquake	0.97	Unstable
	Yes	Normal	1.15	Basic stable
		Rainstorm	1.07	Basic stable
		Earthquake	1.11	Basic stable
2–2′	No	Normal	1.02	Basic stable
		Rainstorm	0.85	Unstable
		Earthquake	0.95	Unstable
	Yes	Normal	1.14	Basic stable
		Rainstorm	1.06	Basic stable
		Earthquake	1.10	Basic stable
3–3′	No	Normal	1.05	Basic stable
		Rainstorm	0.88	Unstable
		Earthquake	0.98	Unstable
	Yes	Normal	1.17	Basic stable
		Rainstorm	1.08	Basic stable
		Earthquake	1.12	Basic stable

## Engineering control measure

Based on findings on the landslide characteristics, the main engineering control measure for stabilizing the landslide is to install anti-sliding piles because there already exists a drainage system in the proximity of the landslide (Fig. 4). Anti-sliding piles, preferred by many engineers, are considered as an effective remedial measure in landslide control projects^29, 30^. The anti-sliding piles provide resisting forces to maintain the slope stability, its retaining effect is prominent, its structure is simple, and its design theory is relatively mature. The design calculations of the anti-sliding pile involve four aspects^31–33^: (1) calculation of the thrust force; (2) calculation of the pile stability; (3) calculation of the internal force; and (4) calculation of the longitudinal bar and stirrup.

The thrust force acting on the cantilever part of the anti-sliding pile above the slip surface can be expressed as follows:

10 $$P_{i} = P_{i - 1} \times \psi + K_{s} \times T_{i} - R_{i}$$

11 $$T_{i} = W_{i} (\sin \alpha_{i} + A\cos \alpha_{i} ) + \gamma_{W} h_{{i_{W} }} L_{i} \cos \alpha_{i} \sin \beta_{i} \cos (\alpha_{i} - \beta_{i} )$$

12 $$R_{i} = (W_{i} (\cos \alpha_{i} - A\sin \alpha_{i} ) - \gamma_{W} h_{{i_{W} }} L_{i} - \gamma_{W} h_{{i_{W} }} L_{i} \cos \alpha_{i} \sin \beta_{i} \sin (\alpha_{i} - \beta_{i} ))\tan \varphi_{i} + C_{i} L_{i}$$

13 $$\psi = \cos (\alpha_{{i{ - }1}} - \alpha_{i} ) - \sin (\alpha_{{i{ - }1}} - \alpha_{i} )\tan \varphi_{i}$$

where *P*_*i*_ is the thrust force of the *i*th calculation block; *P*_*i*-1_ is the residual sliding force of the *i*th calculation block; *K*_*s*_ is the desired safety factor; *T*_*i*_ is the sliding force; and *R*_*i*_ is the resisting force.

The calculation on the pile stability can be obtained as follows:

14 $$\sigma_{\max } \le P_{r} \times (\sigma_{p} - \sigma_{a} )$$

where σ_max_ is the maximum lateral pressure of the embedded part; *Ρ*_*r*_ is the reduction factor; σ_*p*_ is the passive earth pressure; and σ_*a*_ is the active earth pressure.

The calculation of the internal force for the pile is given by:

15 $$K = m(y + y_{0} )^{n}$$

where *K* is the elastic resistance coefficient of the foundation; *m* is the proportion coefficient; *y* is the distance between the embedded part and the slip surface; *y*_0_ is a constant related to geotechnical properties; and *n* is a geotechnical material constant.

The calculation of the longitudinal bar and stirrup for the pile can be expressed as follows:

16 $$A_{s} = \frac{{K_{1} M}}{{\gamma_{s} f_{y} h_{0} }}$$

17 $$\gamma_{s} = \frac{{1 + \sqrt {1 - 2a_{s} } }}{2}$$

18 $$a_{s} = \frac{{K_{1} M}}{{f_{cm} bh_{0}^{2} }}$$

19 $$V_{cs} = 0.07f_{c} bh_{0} + 1.5f_{yv} \frac{{A_{sv} }}{S}h_{0}$$

20 $$K_{2} V \le 0.25f_{c} bh_{0}$$

where *A*_*s*_ is the cross-sectional area of the longitudinal bar; *M* is the design bending moment of the anti-sliding pile; *f*_*y*_ is the design value of the tensile strength of the longitudinal bar; *f*_*cm*_ is the design value of the compressive strength of concrete; *h*_0_ is the effective height of the pile section; *b* is the width of the pile section; *K*_1_ is the desired safety factor of the bending strength of the pile; *V* is the design value of the shear force of the pile; *V*_*cs*_ is the shear bearing capacity of concrete and stirrup of the pile section; *f*_*c*_ is the design value of the concentric axial compression of concrete; *f*_*yv*_ is the stirrups’ tensile characteristic strength; *A*_*sv*_ is the cross-sectional area of stirrups crossing the pile section; *S* is the stirrup spacing; and *K*_2_ is the desired safety factor of the shear strength of the pile.

**Figure 9:**
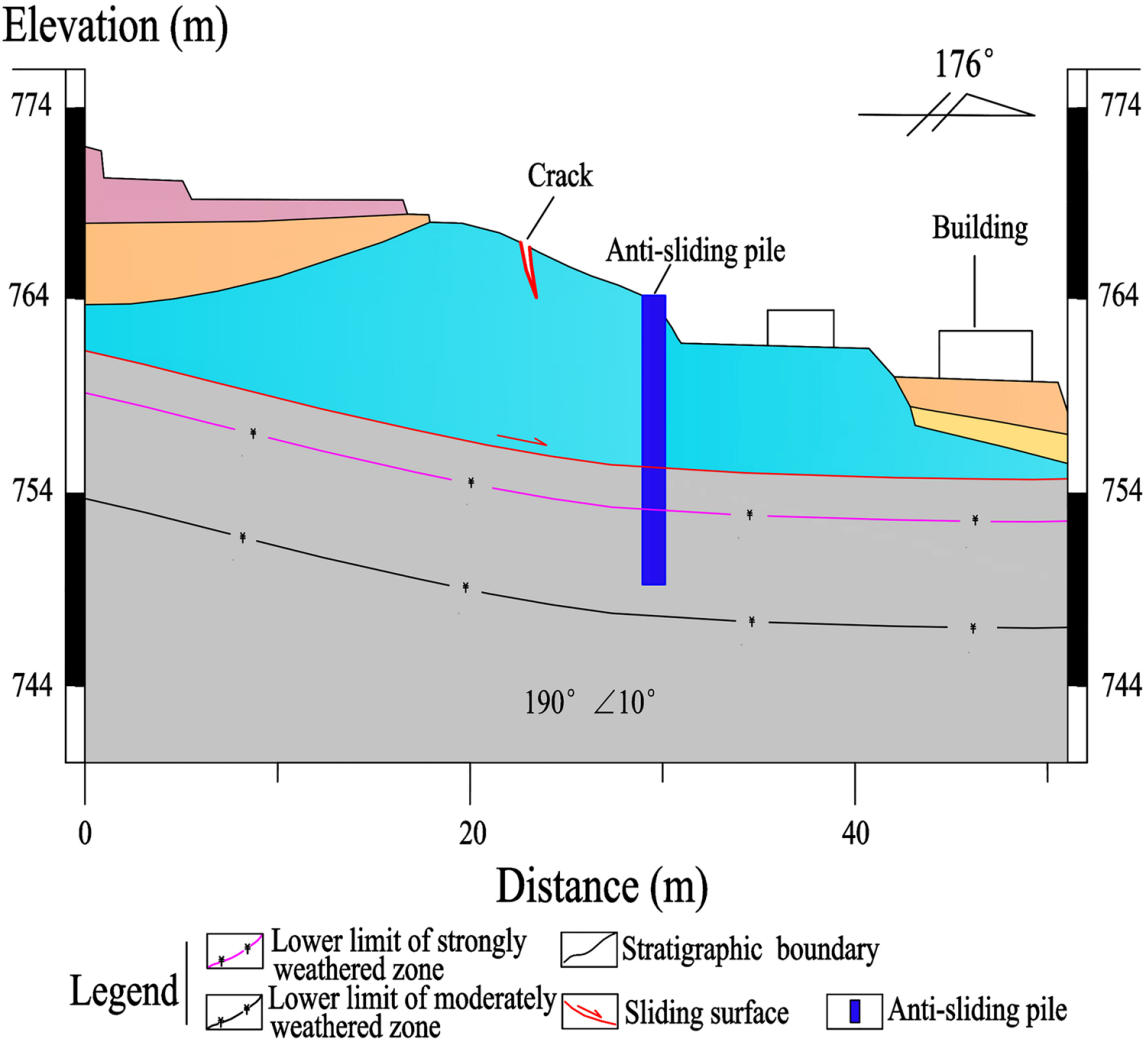
Longitudinal geological profile 4–4′ (Fig. 4).

**Figure 10:**
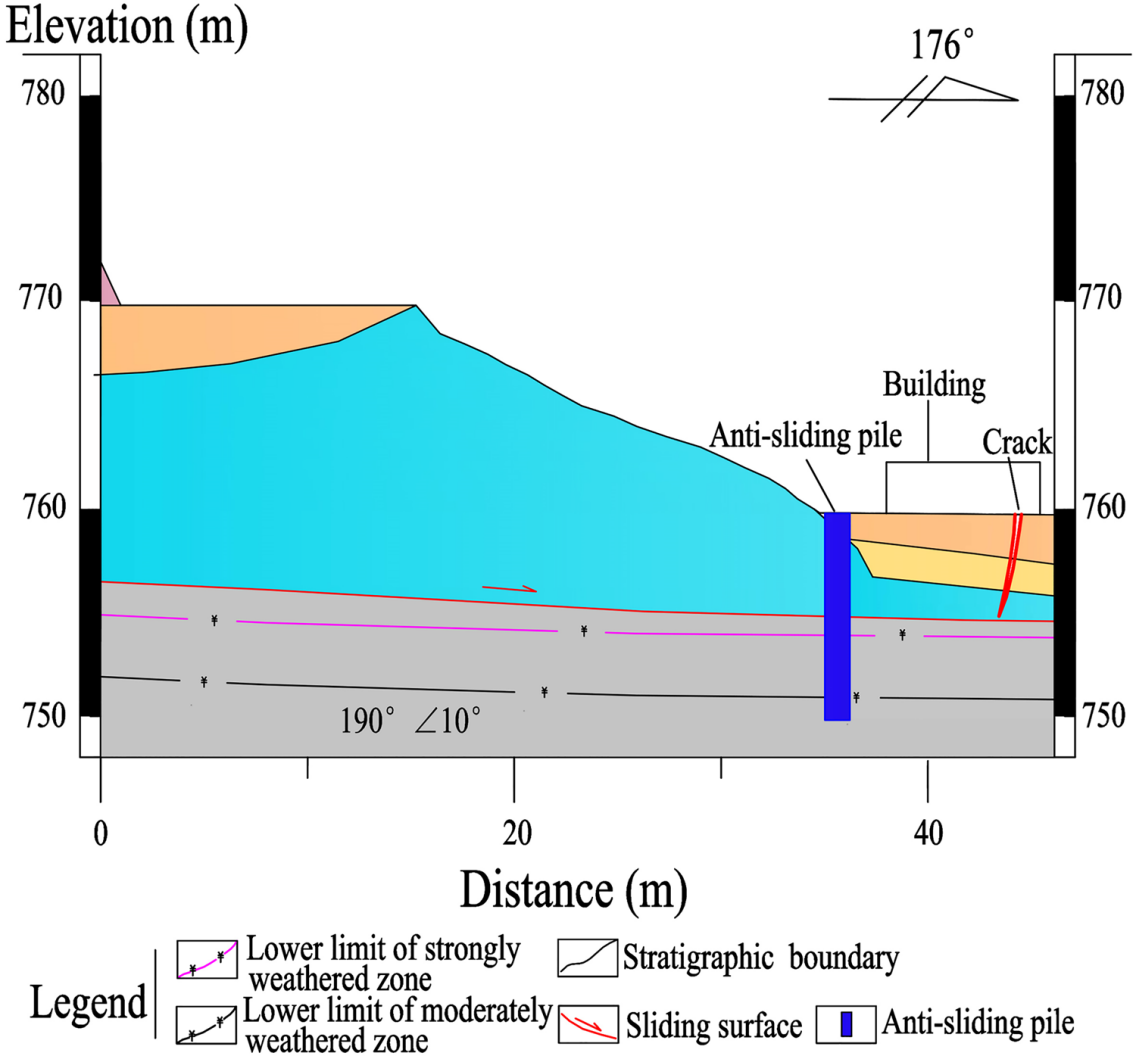
Longitudinal geological profile 5–5′ (Fig. 4).

**Figure 11:**
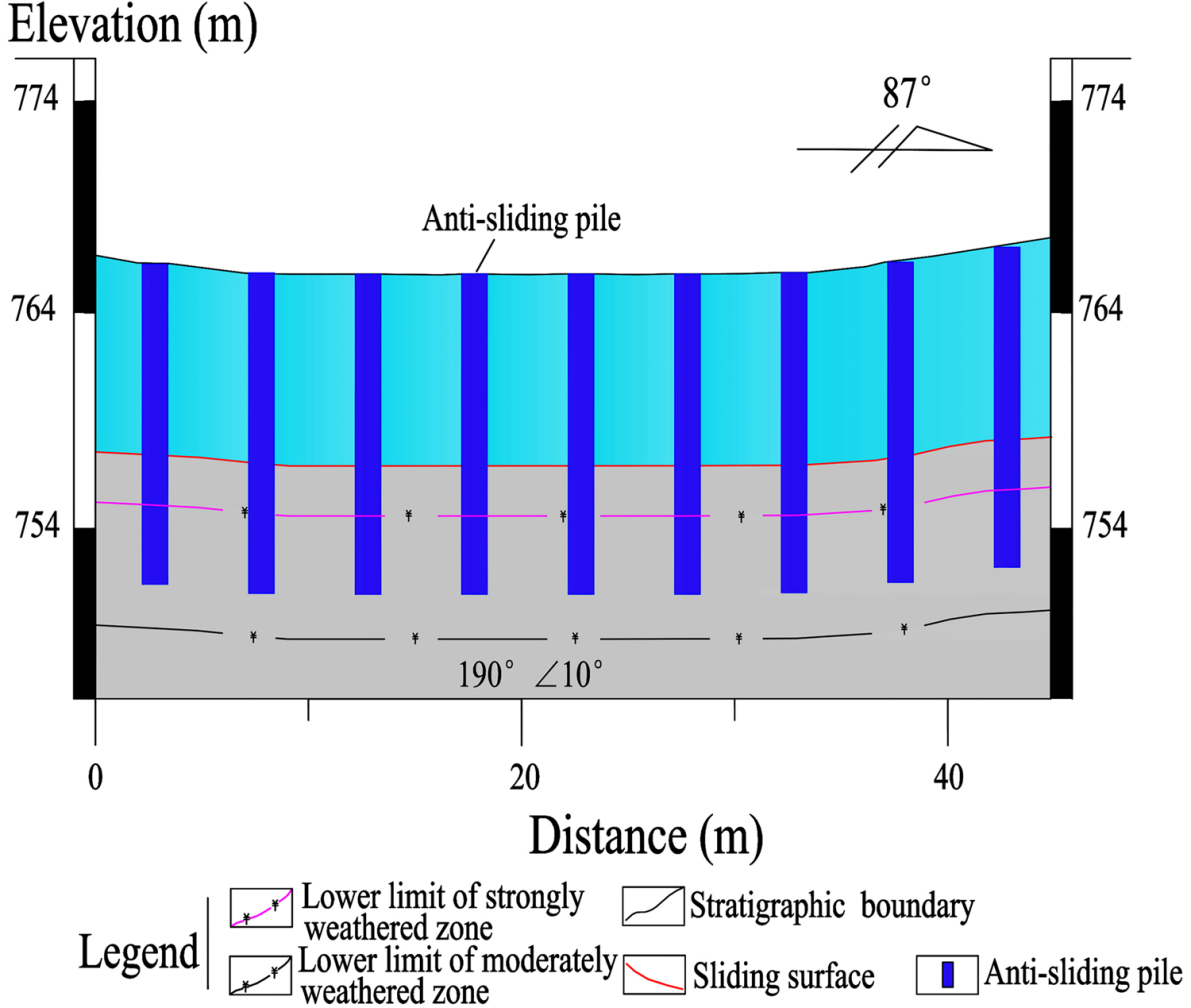
Longitudinal geological profile 6–6′ (Fig. 4).

**Figure 12:**
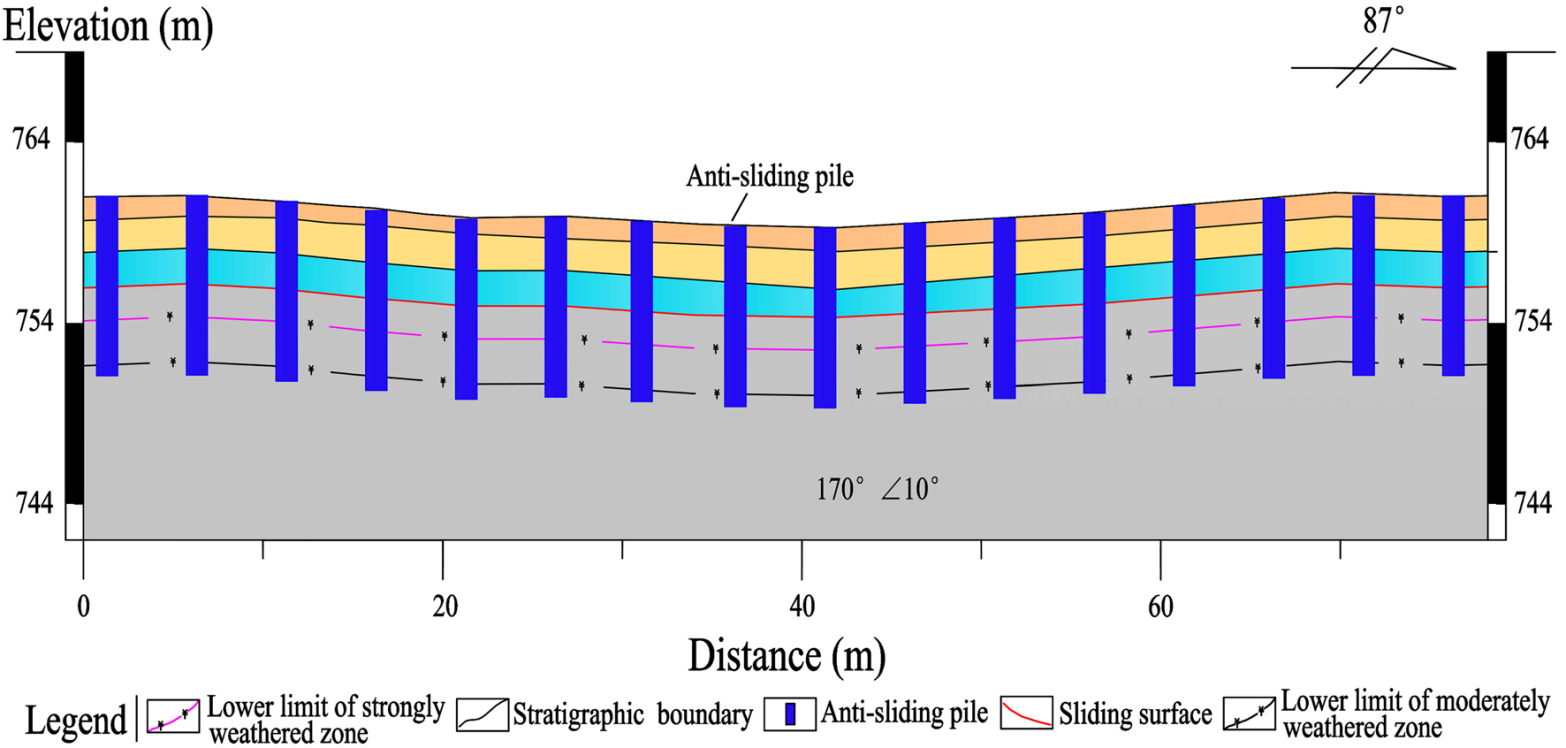
Longitudinal geological profile 7–7′ (Fig. 4).

A total of 25 anti-sliding piles, with a rectangular cross-section of 1.2 × 1.5 m and a length of 10–15 m, are constructed in the middle-lower section of the Laochang landslide (Figs. 9, 10, 11, 12). The center distance between the two adjacent piles is 5.0 m, and the embedment depth of each pile into the bedrock is 4.5–6.5 m. The Laochang landslide with anti-sliding piles would become stable under rainstorm or earthquake conditions (Table 3).

**Figure 13:**
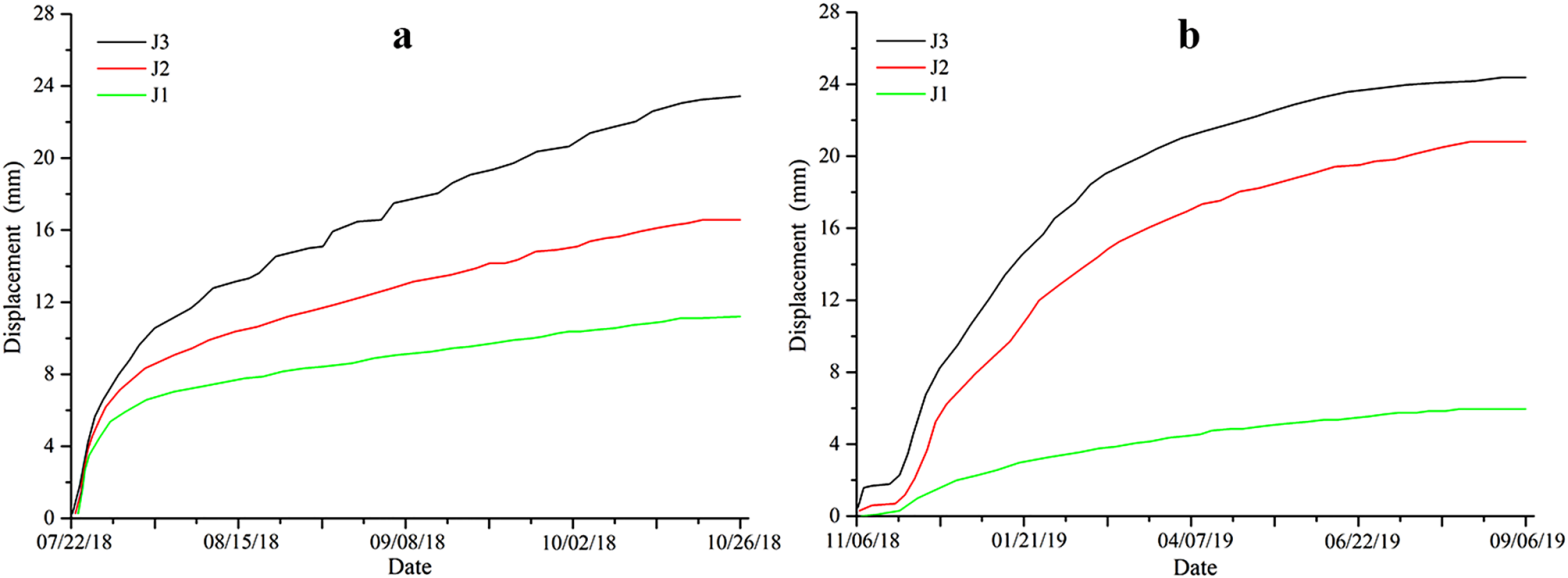
Surface displacement monitoring result: (**a**) before the construction of anti-sliding piles; (**b**) after the construction of anti-sliding piles (Fig. 4).

Figure 13 shows that anti-sliding piles significantly reduced the rates of surface displacement. For the monitoring point J3, the rate of surface displacement after construction was 0.079 mm/day, in contrast to 0.244 mm/day before the construction of anti-sliding piles. For the monitoring point J2, the rate of surface displacement after the construction of anti-sliding piles was 0.068 mm/day, compared with 0.173 mm/day before construction. Before the construction of anti-sliding piles, surface displacement increased rapidly during the rainy season. After the construction of anti-sliding piles, no significant, sudden increased displacement was observed during the rainy season. The displacement curves were relatively flat and the rates of surface displacement were also small. This indicated that anti-sliding piles had a remarkable effect on slope stability. From Fig. 13b, the displacement curves of the two monitoring points (J2 and J3) increased rapidly from November 2018 to January 2019. The main reason for this increase was the disturbance of the engineering construction between the two rows of the anti-slide piles.

**Figure 14:**
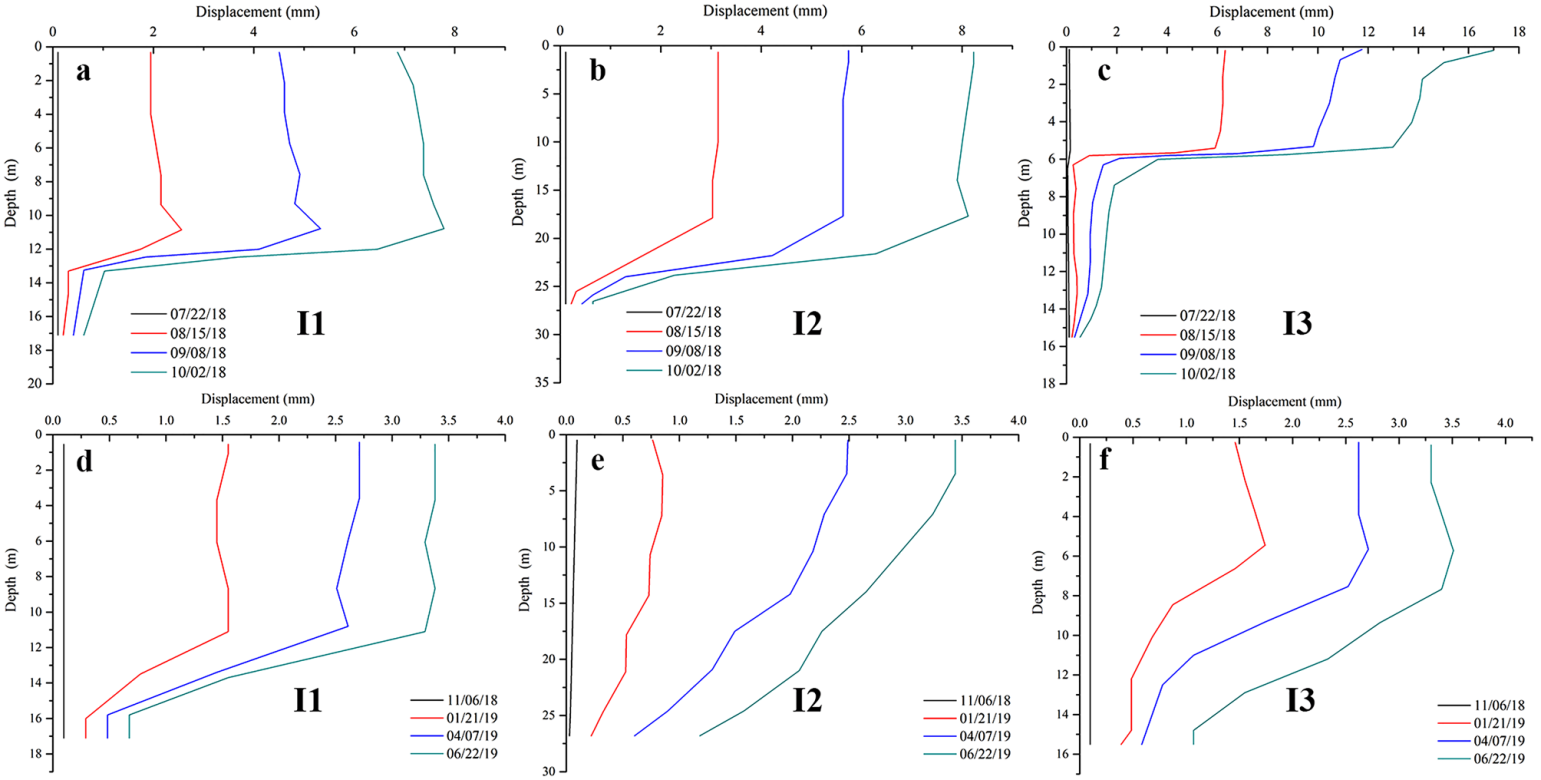
Deep displacement obtained by inclinometers: (**a**–**c**) before the construction of anti-sliding piles; (**d**–**f**) after the construction of anti-sliding piles (Fig. 4).

To further determine the stability of the slope, it was necessary to conduct a deep displacement monitoring project before and after the construction of anti-sliding piles. Three inclinometers, including I1, I2, and I3, were installed along the sliding direction (Fig. 4). The displacements in the lower part of the landslide (I3) were larger than that in the middle (I2) and upper (I1) areas (Fig. 14a–c), indicating that the deformation of the lower part of the landslide is more pronounced than the deformations of other parts. As shown in Fig. 14d–f, the slope reinforced by the anti-slide piles was in a stable state, thereby indicating the positive effect of the anti-slide piles. For the inclinometer I3, the displacement rate after the construction of anti-sliding piles was 0.015 mm/day, compared with 0.236 mm/day before construction. For I2, the displacement rate after construction was 0.015 mm/day, in contrast to 0.114 mm/day before the construction of anti-sliding piles. For I1, the displacement rate decreased from 0.108 to 0.014 mm/day after the construction of anti-sliding piles. Both surface and deep displacement increases were significantly reduced for all the monitoring points by the presence of anti-sliding piles. The reinforcement effect of anti-slide piles on the slope deformation was obviously positive, and the slope body gradually became stable.

## Conclusions

This paper reported that the Laochang landslide occurred on July 15, 2018, in Tianquan County, Sichuan Province, China. Ground investigations, drilled boreholes, exploratory trenches, and displacement monitoring were carried out to investigate the landslide characteristics and to analyze the engineering control measure. The conclusions are as follows:

1. The Laochang landslide has experienced slow creep deformation in the last 30 years and is a slow-moving landslide in the low mountains area, with a volume of 3.15 × 10^5^ m^3^. The slip surface was mainly the interface between the superficial deposits and the weathered bedrock strata. The deformation of the Laochang landslide could be divided into two parts: the front strong deformation part with obvious deformation (such as tension cracks and bulges) and the rear weak deformation part with no obvious large deformation but some small surface fissures formed during the rainy season.
2. At present, the landslide still remains unstable, potentially endangering the lives and properties of residents downslope. Therefore, the main engineering control measure such as slope stabilizing piles was installed to strengthen the sliding body above the slip surface by placing the piles embedded into the bedrock. After the construction of the anti-sliding piles, the surface and deep displacement of the landslide was reduced significantly for all the monitoring points, indicating the effectiveness of anti-sliding piles in reducing the risk of the landslide.

## Data Availability

The raw/processed data required to reproduce these findings cannot be shared at this time as the data also forms part of an ongoing study.
